# P62 in colorectal cancer: from inflammation suppression to cancer promotion

**DOI:** 10.3389/fimmu.2026.1760631

**Published:** 2026-03-25

**Authors:** Niumuqie A, Jianlin Huang, Lin Liu, Hui Chen, Jiaxin Chen, Shuntian Zou, Yuxuan Pang, Xu Wu, Minghua Liu, Gang Liang

**Affiliations:** 1 School of Pharmacy, Southwest Medical University, Luzhou, China; 2 Department of Pharmacy, Luzhou Naxi District People’s Hospital, Luzhou, Sichuan, China; 3 Drug Dispending Department, The Third Hospital of Mianyang, Sichuan Mental Health Center, Mianyang, China; 4 Department of Pediatrics, Southwest Medical University, Luzhou, China; 5 Department of Pharmacy, The Affiliated Traditional Chinese Medicine Hospital, Southwest Medical University, Luzhou, China

**Keywords:** autophagy, colorectal cancer, DNA damage repair, inflammation suppression, mTORC1, NF-κB, Nrf2, p62

## Abstract

The autophagy adaptor and scaffold protein p62 plays a critical role in colorectal cancer (CRC) progression. As a malignancy driven by chronic inflammation, CRC arises from the combined effects of oncogenic signaling activation, genetic mutations, and epithelial barrier disruption. During early stages of inflammation, p62 exerts tumor-suppressive functions by clearing inflammasomes, activating NRF2, and downregulating inflammatory cytokines. However, sustained inflammation leads to p62 accumulation, which reinforces the activation of NRF2, NF-κB, and mTORC1 pathways through positive feedback loops, thereby driving tumorigenesis. Excessive p62 also impairs DNA double-strand break repair, facilitating oncogenic mutations. These observations indicate that enhancing autophagic clearance of p62 could represent a promising therapeutic strategy to prevent inflammation-associated CRC.

## 1 Introduction

Colorectal cancer (CRC) is one of the most prevalent malignancies globally, ranking as the third most commonly diagnosed cancer and the second leading cause of cancer-related deaths, representing a significant health burden. The persistently high incidence and mortality rates of CRC are primarily attributed to lifestyle-related risk factors, such as excessive consumption of processed foods and sugar-sweetened beverages, rising obesity rates, and unhealthy behaviors like alcohol use and smoking. The highest incidence rates are observed in countries with a high Human Development Index (HDI), largely due to these dietary and lifestyle patterns. However, as urbanization and Westernization continue, CRC incidence is steadily rising in low- and middle-HDI countries as well (1–3).

As a prototypical inflammation-driven cancer, chronic inflammation plays a central role in CRC pathogenesis (3–6). Persistent inflammation facilitates immune cell infiltration into tissues, creating a tumor microenvironment (TME) conducive to cancer initiation (7). These immune cells secrete various cytokines that exacerbate inflammation while simultaneously activating multiple pro-tumorigenic signaling pathways, thereby promoting cancer cell proliferation (8, 9). Additionally, the accumulation of reactive oxygen species (ROS) and reactive nitrogen species (RNS) during chronic inflammation can damage cellular genetic material, and the cumulative genetic damage over time leads to mutations (9, 10). Although only 1–2% of patients with inflammatory bowel disease (IBD), including ulcerative colitis (UC) or Crohn’s disease (CD), progress to IBD-associated CRC (IBD-CRC), the mortality rate remains extremely high, accounting for approximately 15% of IBD-related deaths. Long-term epidemiological studies have shown that patients with IBD face a significantly increased risk of developing CRC (2). Throughout the entire process, from the onset of chronic inflammation to malignant transformation, the involvement of p62 protein is evident. p62, a multifunctional scaffold protein and a well-established selective autophagy adaptor, possesses multiple functional domains that enable interactions with various cellular partners, regulating key processes such as redox homeostasis, inflammation, metabolism, and DNA damage repair (11). During the early stages of CRC-associated inflammation, p62 mitigates inflammatory activation and promotes the clearance of pro-inflammatory cytokines through both autophagy-dependent and autophagy-independent mechanisms, thereby exerting a tumor-suppressive effect (12, 13). However, with prolonged inflammation in the intestinal epithelium, p62’s pro-tumorigenic functions gradually predominate. Continuous activation of proliferative signaling pathways, such as Nrf2 and NF-κB, which are upregulated during inflammation and cancer, leads to increased p62 expression, as both are transcriptional targets of these pathways (14–16). In turn, p62, through its TRAF6-binding (TB) and LIM protein-binding (LB) domains, interacts with associated proteins to further activate Nrf2 and NF-κB (17–19), establishing positive feedback loops that drive tumor cell proliferation. Additionally, p62 activates the mechanistic target of rapamycin (mTOR) via its TB domain, simultaneously inhibiting autophagy and enhancing mTOR-mediated pro-survival and proliferative signaling (20–23). Impaired autophagic degradation further contributes to p62 accumulation, reinforcing these feedback loops (24, 25). Sustained elevation of intracellular p62, particularly its nuclear accumulation, disrupts DNA damage repair, leading to genetic alterations that drive oncogenic mutations and promote CRC progression (26). Collectively, p62 plays a dual role in CRC: it suppresses inflammation and prevents CRC onset during the early stages but shifts to a tumor-promoting role with sustained intracellular accumulation. Notably, significant p62 accumulation has been observed in CRC (27–29). And multiple clinical studies have shown that p62 overexpression is associated with the prognosis and tumor stage of CRC patients (**Table 1**).

**Table 1:** Clinical studies on p62 expression in patients with CRC.

Study	Sample size	Method	Main findings	Reference
Study 1	85 CRC	HIC RT-qPCR	P62 is aberrantly overexpressed in tumor tissues and correlates with advanced stage and lymph node metastasis, independently predicting poor Progression-free survival (PFS) and overall survival (OS)	(27)
Study 2	146 CRC	HIC	High p62 expression is significantly associated with liver metastasis in CRC and has been identified as an independent adverse prognostic factor in patients with CRC.	(138)
Study 3	127 CRC	HIC	Cytoplasmic p62 expression is significantly associated with overall survival (OS) in CRC patients harboring KRAS mutations.	(139)
Study 4	129 CRC	HIC	P62 expression is significantly higher in colon cancer tissues than in normal tissues and is associated with patient overall survival (OS).	(140)
Study 5	92 CRC	IHC qPCR	P62 mRNA is markedly upregulated in tumor tissues, and high p62 expression is significantly associated with advanced lymph node metastasis and reduced overall survival (OS).	(141)
Study 6	601 CRC	IHC	P62 expression levels are associated with the tumor immune microenvironment and may play a role in immune regulation in CRC.	(142)
Study 7	47 CRC	IHC	Higher p62 expression is significantly associated with reduced chemoradiotherapy (CRT) efficacy in patients with advanced rectal cancer.	(143)

This review first outlines the structural features of p62 and then delves into its molecular mechanisms in CRC pathogenesis, including its role in suppressing CRC-associated inflammation, its cytoplasmic accumulation through inflammation-related pathways and autophagy inhibition, its activation of key oncogenic signaling cascades (NF-κB and mTOR), and its contribution to CRC progression via interference with DNA damage repair. Finally, this review discusses the feasibility of reducing CRC risk and inhibiting its progression by promoting autophagy in intestinal epithelial cells to clear excessive accumulation of p62 protein.

## 2 Basic structure of p62

**Figure 1:**
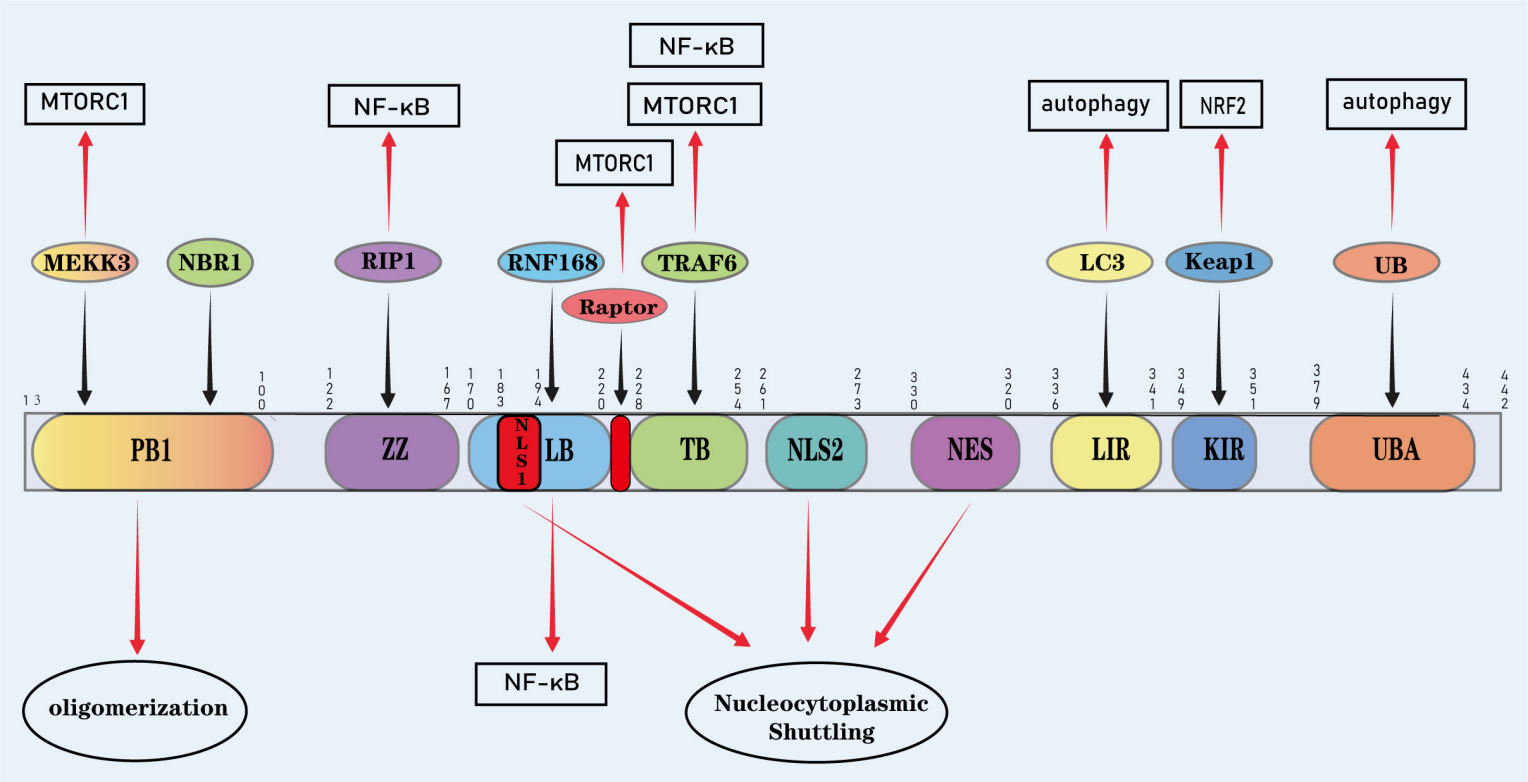
Basic structure of P62 protein: The functional domains of P62 underpin its roles in various processes. The N-terminal Phox1 and BP1 domains mediate self-oligomerization and regulate processes like NF-κB activation via MEKK3 binding. BP1 also interacts with the autophagy receptor NBR1. The ZZ-type zinc finger (ZZ) domain activates NF-κB through RIP binding. The LIM-binding (LB) domain interacts with E3 ligase RNF168 to regulate mTORC1 and NF-κB. The Raptor interaction region between LB and TB domains also aids mTORC1 activation. Two nuclear localization signals (NLS) and one nuclear export signal (NES) control P62’s nuclear localization and shuttling. The LC3-interacting region (LIR) allows P62 to function as an autophagic cargo receptor. The KIR domain enhances Nrf2 activity by interacting with Keap1. Finally, the C-terminal UBA domain interacts with ubiquitinated substrates in the autophagy-UPS pathway to guide cargo for lysosomal degradation. MEKK3, Mitogen-Activated Protein Kinase 3; NBR1, Neighbor of BRCA1 Gene; RIP1, Receptor-Interacting Protein Kinase 1; RNF168, Ring Finger Protein 168; Raptor, Regulatory-Associated Protein of mTOR; TRAF6, TNF Receptor-Associated Factor 6; LC3, Microtubule-Associated Protein 1 Light Chain 3; Keap1; Kelch-Like ECH-Associated Protein 1; UB, Ubiquitin.

The scaffold protein and stress-inducible autophagy adaptor p62, also known as sequestosome-1 (SQSTM1), is encoded by the human *SQSTM1* gene located on chromosome 5. This gene is evolutionarily conserved across metazoans and comprises eight exons spanning approximately 16 kb (13, 30). Functionally, p62 serves as both a receptor and shuttle protein, linking intracellular cargo to degradation pathways via the proteasome and autophagy. Additionally, p62 is involved in regulating multiple signaling pathways, with these diverse functions stemming from its structural characteristics. The protein consists of 440 amino acids organized into several functional domains, each facilitating specific protein–protein interactions and downstream effects, forming the molecular foundation for its biological roles (**Figure 1**) (31).

At the N-terminus, the Phox and Bem1p (PB1) domain mediates p62’s self-oligomerization (31), a property critical for clustering selective autophagy cargo and promoting its degradation. Beyond self-assembly, the PB1 domain interacts with other PB1-containing proteins, such as members of the mitogen-activated protein kinase (MAPK) signaling module—including MEKK2, MEKK3, and MEK5—thereby regulating various physiological and cellular processes (32, 33). For example, the PB1 domain’s interaction with MEKK3, a component of the AMP-activated protein kinase (AMPK) signaling module, plays a role in modulating NF-κB activation (34). Moreover, p62 utilizes its PB1 domain to interact with the selective autophagy receptor next to BRCA1 gene 1 (NBR1). NBR1 shares structural similarity with p62, possessing domains that enable direct binding to both ATG8 family proteins and ubiquitin. In mammals, NBR1 is involved in the degradation of protein aggregates, peroxisomes, midbody remnants, focal adhesions, and major histocompatibility complex class I (MHC-I) receptors (32, 35). Following the PB1 domain, the ZZ-type zinc finger (ZZ) domain of p62 binds to the tumor necrosis factor receptor-interacting protein (RIP). Members of the atypical protein kinase C (aPKC) subfamily, including PKCζ and PKCλ/ι, regulate NF-κB activation via IκB kinase gamma (IKKγ). Through its ZZ domain, p62 physically links aPKCs to Receptor-Interacting Protein (RIP). In turn, aPKCs connect IKKγ to p62, forming a multiprotein signaling cascade comprising TNF Receptor-Associated Death Domain (TRADD), RIP, p62, aPKC, and IKKγ, which collectively facilitates NF-κB activation (19).

The LIM protein-binding (LB) domain of p62 further regulates NF-κB signaling by scaffolding a multiprotein complex comprising atypical protein kinase C (aPKC) and the E3 ubiquitin ligase TNF Receptor-Associated Factor 6 (TRAF6), which mediates IL-1- and TNF-α-induced NF-κB activation (36). Additionally, the LB domain interacts with the E3 ligase RNF168, inhibiting its activity. Nuclear RNF168 is essential for histone H2A ubiquitination and the DNA damage response, and its inhibition prevents the recruitment of DNA repair proteins such as BRCA1, RAP80, and Rad51 to sites of DNA double-strand breaks (DSBs), impairing DSB repair (37).

The TRAF6-binding (TB) domain of p62 plays a pivotal role in regulating mechanistic target of rapamycin complex 1 (mTORC1) activation. By interacting with TRAF6, p62 facilitates the lysosomal translocation of mTORC1, where it is subsequently activated by amino acids within specific lysosomal compartments (20, 38). The interaction between p62 and TRAF6 also contributes to NF-κB activation (39), highlighting the multifunctional role of this domain in coordinating proliferative and inflammatory signaling. Further studies have identified an additional region between the LB and TB domains that interacts with Raptor, further promoting mTORC1 activation (39). The nuclear import and export of p62 are regulated by two nuclear localization signals (NLS1/2) and one nuclear export signal (NES) (40). The LC3-interacting region (LIR) domain of P62 is essential for its function as a cargo receptor in the autophagy pathway. Multiple autophagy effectors, including Microtubule-Associated Protein 1 Light Chain 3 (LC3) itself, bind directly to p62 through the LIR motif, enabling p62 to selectively recognize and deliver cargo to autophagosomes for degradation (24, 41).

Near the C-terminus, the Keap1-interacting region (KIR) motif enables p62 to bind Kelch-like ECH-associated protein 1 (Keap1) directly, thereby enhancing Nrf2 activity (42). Finally, at the extreme C-terminal end, the ubiquitin-associated (UBA) domain mediates interactions between p62 and ubiquitinated substrates in both the autophagy and ubiquitin–proteasome system (UPS) pathways, directing these substrates for degradation. In the autophagy pathway, p62 first binds ubiquitinated cargo via its UBA domain, then uses its LIR domain to interact with autophagy effectors, transferring the cargo to nascent autophagosomes for clearance. Conversely, during UPS-mediated degradation, p62 binds ubiquitinated cargo via the UBA domain but associates with the 19S proteasome through its PB1 domain, facilitating proteasomal degradation (43, 44). Notably, even ubiquitinated proteasomes can serve as substrates, and p62 targets them for selective degradation through the coordinated action of its UBA and LIR domains (43).

## 3 p62 suppresses inflammation activation

### 3.1 Inflammation and colorectal cancer

**Figure 2:**
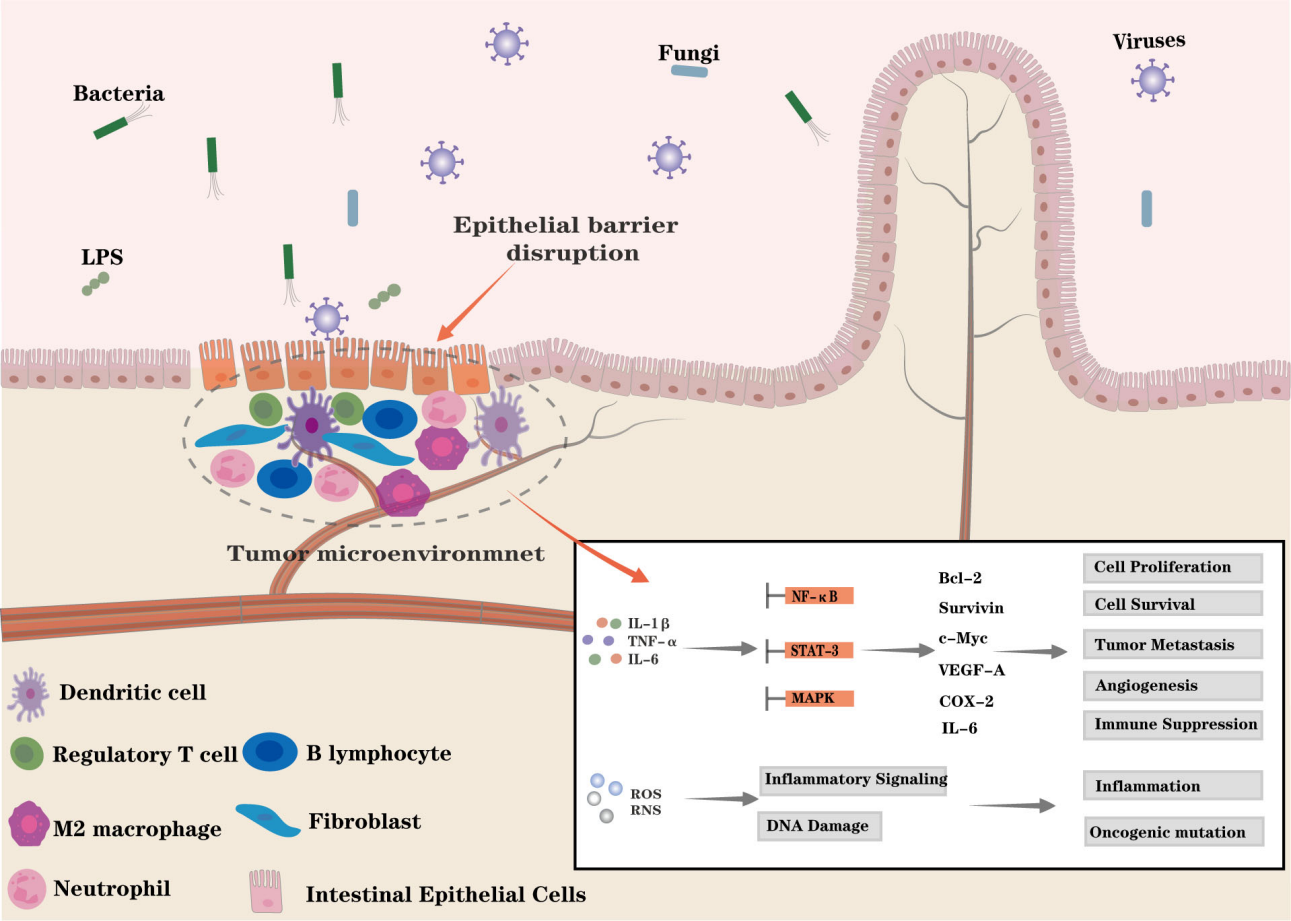
Chronic inflammation promotes colorectal cancer development: Persistent intestinal inflammation drives the formation of a tumor-promoting microenvironment (TME) through interactions among infiltrating immune cells, epithelial cells, and fibroblasts. Infiltrating immune cells secrete inflammatory mediators, including prostaglandins, chemokines, and cytokines (e.g., IL-6, TNF-α, and IL-1β), which activate oncogenic pathways such as IL-6/STAT3, NF-κB, and AMPK. These signals upregulate oncogene expression and increase the levels of their downstream effector proteins (Bcl-2, Cyclin D, c-Myc, Survivin, VEGF6, COX-2 and IL-6), promote angiogenesis, suppress antitumor immunity, and enhance cancer cell survival and metastasis. Tumor-associated macrophages and neutrophils produce reactive oxygen and nitrogen species (ROS/RNS), These reactive species activate diverse inflammatory signaling pathways, including NF-κB and the NLRP3 inflammasome, thereby sustaining inflammatory progression, and they induce DNA damage and genomic instability, increasing the risk of cellular malignant transformation. Persistent inflammation also disrupts epithelial barrier integrity, facilitating microbial translocation and perpetuating inflammation.

Inflammation is a host defense mechanism that involves the activation, recruitment, and function of both innate and adaptive immune cells. Beyond its protective role, inflammation is pivotal for tissue repair, regeneration, remodeling, and maintaining homeostasis (45). However, chronic inflammation is a recognized pro-tumorigenic factor, particularly in the development and progression of CRC (45–47). During chronic inflammation, immune cells, including macrophages, monocytes, neutrophils, and innate lymphoid cells, infiltrate the affected tissues and continuously release inflammatory mediators such as prostaglandins, chemokines, and pro-inflammatory cytokines like IL-6 and TNF-α. These mediators interact with fibroblasts and endothelial cells to establish an inflammatory TME that promotes tumor cell proliferation, invasion, metastasis, angiogenesis, and immune evasion by modulating immune cell metabolism and function to favor tumor growth (**Figure 2**) (10, 48). Cytokines released in this context also activate key oncogenic signaling pathways, including IL-6/STAT3, NF-κB, and MAPK, with the IL-6/STAT3 and NF-κB pathways playing central roles in CRC (47, 49–51). STAT3, activated by IL-6, upregulates several oncogenic genes, including *CCND1* and *MYC* (which promote cell proliferation), *BCL2* and *BIRC5* (which support cell survival), and *VEGFA* (which drives angiogenesis). In addition to inducing oncogenes, STAT3 activation enhances immune suppression by upregulating molecules like IL-10 and PD-L1, facilitating immune evasion. Therefore, the IL-6/STAT3 axis sustains chronic inflammation, promotes tumor cell survival, and supports immune escape, making it crucial for CRC progression (52, 53). Similarly, NF-κB signaling, triggered primarily by pro-inflammatory cytokines (TNF-α, IL-1β, IL-6) and microbial components, is a major driver of CRC. Activated NF-κB induces the expression of genes that promote cell survival and proliferation, notably upregulating *PTGS2*, which increases prostaglandin (PG) production, perpetuating chronic inflammation and tumor growth (51, 54). Persistent NF-κB activation creates a positive feedback loop in which inflammation and tumorigenesis mutually reinforce each other.

A major driver of malignant transformation is the accumulation of mutations and/or epigenetic alterations that inactivate tumor suppressor pathways or activate oncogenic signaling pathways (55). In chronically inflamed tissues, infiltrating macrophages and neutrophils are potent sources of ROS and RNS, which promote genomic instability by inducing DNA damage, impairing normal DNA repair mechanisms, and facilitating replication errors (**Figure 2**). These processes significantly increase the likelihood of malignant transformation (56). Such mechanisms are key drivers of cancer development. For example, mutations in members of the mammalian RAS gene family, such as the Kirsten rat sarcoma viral oncogene homolog (*KRAS*), activate the RAS/MEK signaling pathway, which plays a central role in CRC progression (57). Furthermore, cytokines released by inflammatory cells, including IL-6, TNF-α, and IL-1β, can activate epigenetic mechanisms in epithelial cells. These mechanisms involve alterations in DNA methyltransferases and histone-modifying enzymes, such as DNMT1, DNMT3, and DOT1L, as well as dysregulated expression of non-coding RNAs, including microRNAs (miRNAs) and long non-coding RNAs (lncRNAs), which cooperatively regulate the expression of oncogenes and tumor suppressor genes. The cumulative effect of these epigenetic alterations resembles that of genetic mutations, resulting in functional inactivation of tumor suppressor genes and activation of oncogenes, thereby further promoting the initiation and progression of CRC (58).

In CRC, persistent disruption of the intestinal epithelial barrier is a hallmark of disease progression. Chronic inflammatory conditions, such as CD and UC, further contribute to epithelial barrier damage. The intestinal epithelial barrier, formed by tightly interconnected epithelial cells, serves as a critical physiological interface—it mediates nutrient absorption while preventing harmful luminal antigens and noxious substances from penetrating underlying tissues. Barrier dysfunction exacerbates inflammation by sustaining immune activation and facilitating further immune cell infiltration into inflamed tissues, thereby amplifying the inflammatory response (59, 60). This creates a vicious cycle in which inflammation drives barrier breakdown, and barrier disruption in turn perpetuates inflammation. Ultimately, this reciprocal relationship promotes the malignant transformation of epithelial cells, contributing to CRC development (**Figure 2**) (61).

### 3.2 p62 suppresses intestinal inflammation via autophagy-dependent pathways

Inflammasomes serve as sensors for pathogen-associated molecular patterns (PAMPs) and trigger innate immune inflammatory responses. However, their aberrant activation can lead to excessive inflammation, resulting in severe tissue damage and the development of inflammatory diseases. Inflammasomes play a pivotal role in chronic intestinal inflammation (62, 63). Through autophagy-dependent mechanisms, p62 mitigates inflammation by inhibiting inflammasome activation in macrophages and promoting the degradation of inflammasomes and pro-inflammatory cytokines, thereby limiting the initiation and progression of inflammation.

**Figure 3:**
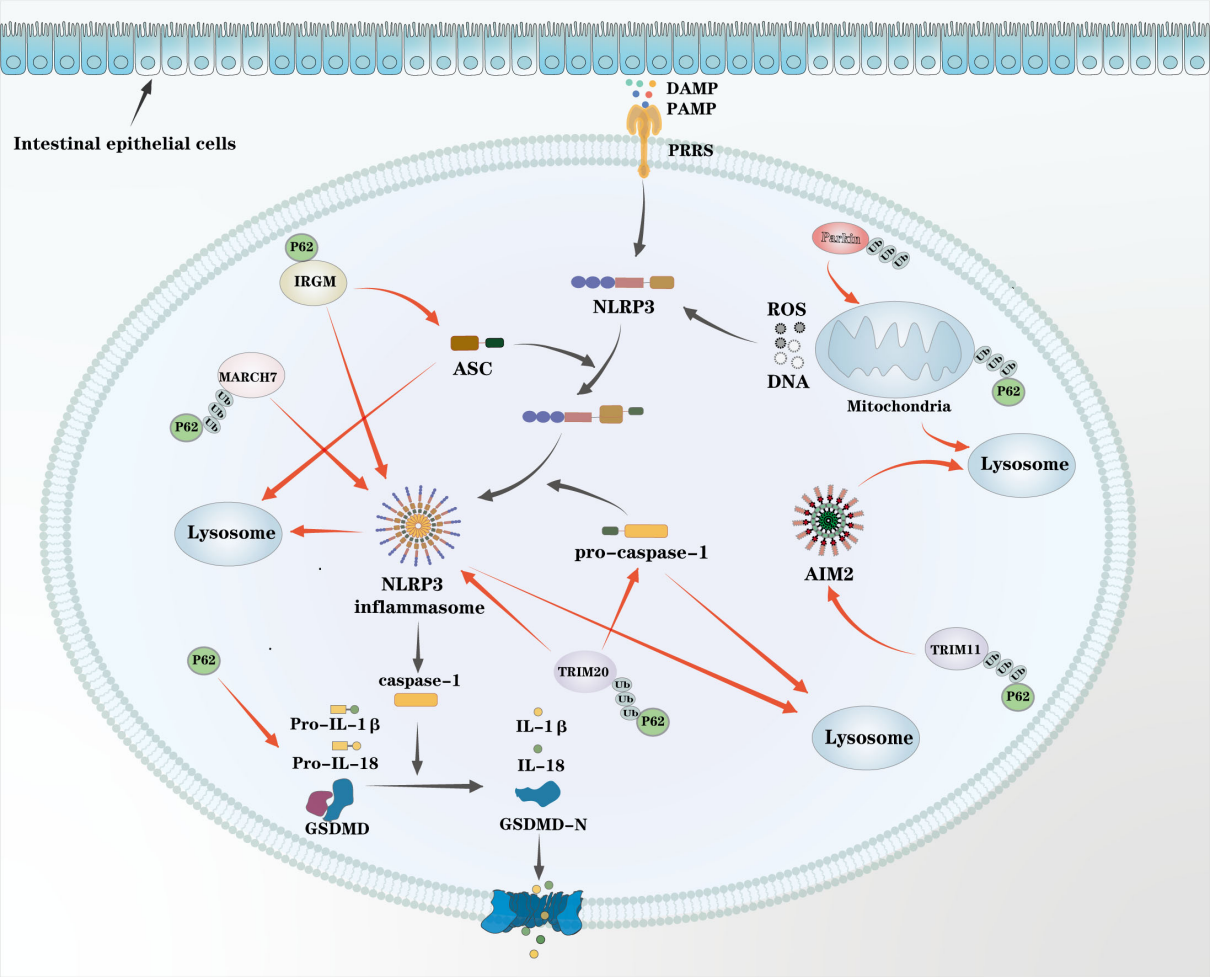
P62 suppresses inflammasome activation: Innate immune pattern recognition receptors (PRRs) detect inflammatory stimuli and activate inflammasome sensors such as NLRP3 and AIM2, leading to ASC oligomerization, caspase-1 activation, and maturation of IL-1β and IL-18. Activated caspase-1 also cleaves gasdermin D (GSDMD), inducing pyroptosis and cytokine release. P62 negatively regulates this process via autophagy. Damaged mitochondria, ubiquitinated by E3 ubiquitin ligase Parkin, are recognized and degraded by P62-dependent mitophagy, preventing NLRP3 activation. Inflammasome components can be ubiquitinated by E3 ligases and degraded via P62-mediated autophagy, such as TRIM20 ubiquitinates NLRP3 and pro–caspase-1, TRIM11 targets AIM2, and MARCH7 modifies NLRP3. The immune-related GTPase IRGM further recruits P62 to promote autophagic degradation of NLRP3 and ASC. In addition, P62 facilitates autophagic clearance of downstream inflammatory mediators, including pro–IL-1α and pro–IL-1β. P62 also mediates the lysosomal degradation of damaged mitochondria, thereby preventing the excessive production of reactive oxygen and nitrogen species.

Innate immune pattern recognition receptors (PRRs) detect PAMPs and damage-associated molecular patterns (DAMPs) to initiate inflammatory responses. PRRs are generally classified into five major families based on their receptor proteins: Toll-like receptors (TLRs) and C-type lectin receptors (CLRs), which recognize extracellular PAMPs, DAMPs, or endosomal ligands; cytosolic RIG-I-like receptors (RLRs); AIM2-like receptors (ALRs); and nucleotide-binding domain leucine-rich repeat-containing receptors (NLRs), which sense cytosolic PAMPs and DAMPs (64). Among these, NLRs and ALRs are particularly pivotal in chronic intestinal inflammation, as they drive the assembly of inflammasomes (65, 66). Inflammasomes are typically composed of a cytosolic NLR or ALR sensor, the adaptor protein apoptosis-associated speck-like protein containing a caspase activation and recruitment domain (ASC), and cysteine-aspartic protease 1 (Caspase-1). Upon activation, NLRs or ALRs recruit ASC, which subsequently activates Caspase-1. Activated Caspase-1 cleaves pro-IL-1β and pro-IL-18 into their mature forms, IL-1β and IL-18, both of which are key pro-inflammatory cytokines involved in regulating various aspects of the inflammatory response and driving disease progression (63). Additionally, Caspase-1 cleaves gasdermin D (GSDMD) to release its N-terminal fragment, which oligomerizes to form pores in the plasma membrane, causing cell swelling, rupture, and pyroptotic cell death. Pyroptosis further enhances the release of IL-1β and IL-18, thereby amplifying the inflammatory cascade (**Figure 3**) (67). NOD-like receptor family pyrin domain containing 3 (NLRP3) is the prototypical inflammasome, activated not only by PAMPs and DAMPs but also by microbial infections, crystalline or particulate matter, and extracellular ATP. These stimuli often induce mitochondrial damage, leading to the release of endogenous ‘mitochondrial DAMPs, including oxidized mtDNA, dsDNA, dsRNA, cardiolipin, and ROS, which are potent activators of NLRP3. Among these, mitochondrial ROS and mtDNA are considered critical signals for inflammasome activation (68). p62 plays a pivotal role in mitophagy: damaged mitochondrial membranes are ubiquitinated by the E3 ligase Parkin and subsequently recognized by p62, which delivers them for autophagic degradation. Through this mechanism, p62 limits the availability of mitochondrial DAMPs, thereby reducing NLRP3 inflammasome activation and suppressing inflammation (**Figure 3**) (69, 70).

Beyond mitochondrial clearance, p62 also acts as a selective autophagy adaptor, directly targeting ubiquitinated inflammasomes and their components for degradation (**Figure 3**). Inflammasome activation itself can trigger autophagy in macrophages via RalB-mediated signaling. Specifically, the small GTPase RalB and its effector Exo84 promote the assembly of the ULK1 and Beclin1–VPS34 complexes at membranes, initiating autophagosome formation (71). Inflammasome components undergo ubiquitination by various E3 ligases, after which they are recognized by p62 and directed to autophagosomes. Tripartite motif-containing proteins (TRIM proteins) are central to this process: TRIM20 ubiquitinates NLRP3 and pro-Caspase-1, enabling their interaction with p62 and subsequent degradation, while TRIM11 targets AIM2 in a similar manner (72–74). Additionally, the E3 ligase MARCH7 ubiquitinates NLRP3, and the immunity-related GTPase M (IRGM) directly recruits p62 to promote the autophagic degradation of NLRP3 and ASC (75, 76).

In addition to suppressing inflammasome activation, p62 functions as a versatile autophagy adaptor to eliminate damaged organelles such as lysosomes, endoplasmic reticulum, and peroxisomes, as well as intracellular bacteria and viruses. By facilitating the selective autophagic degradation of these potential sources of danger signals, p62 prevents the accumulation of pro-inflammatory stimuli, thereby restraining the initiation of inflammation (77).

### 3.3 P62 directly activates the key inflammation-regulating protein Nrf2

Beyond its autophagy-dependent role in removing inflammasome components and other pro-inflammatory factors to suppress inflammation, P62 also promotes Nrf2 activation through ubiquitination, thereby modulating the transcriptional regulation of inflammation-related genes and exerting anti-inflammatory effects (78, 79).

**Figure 4:**
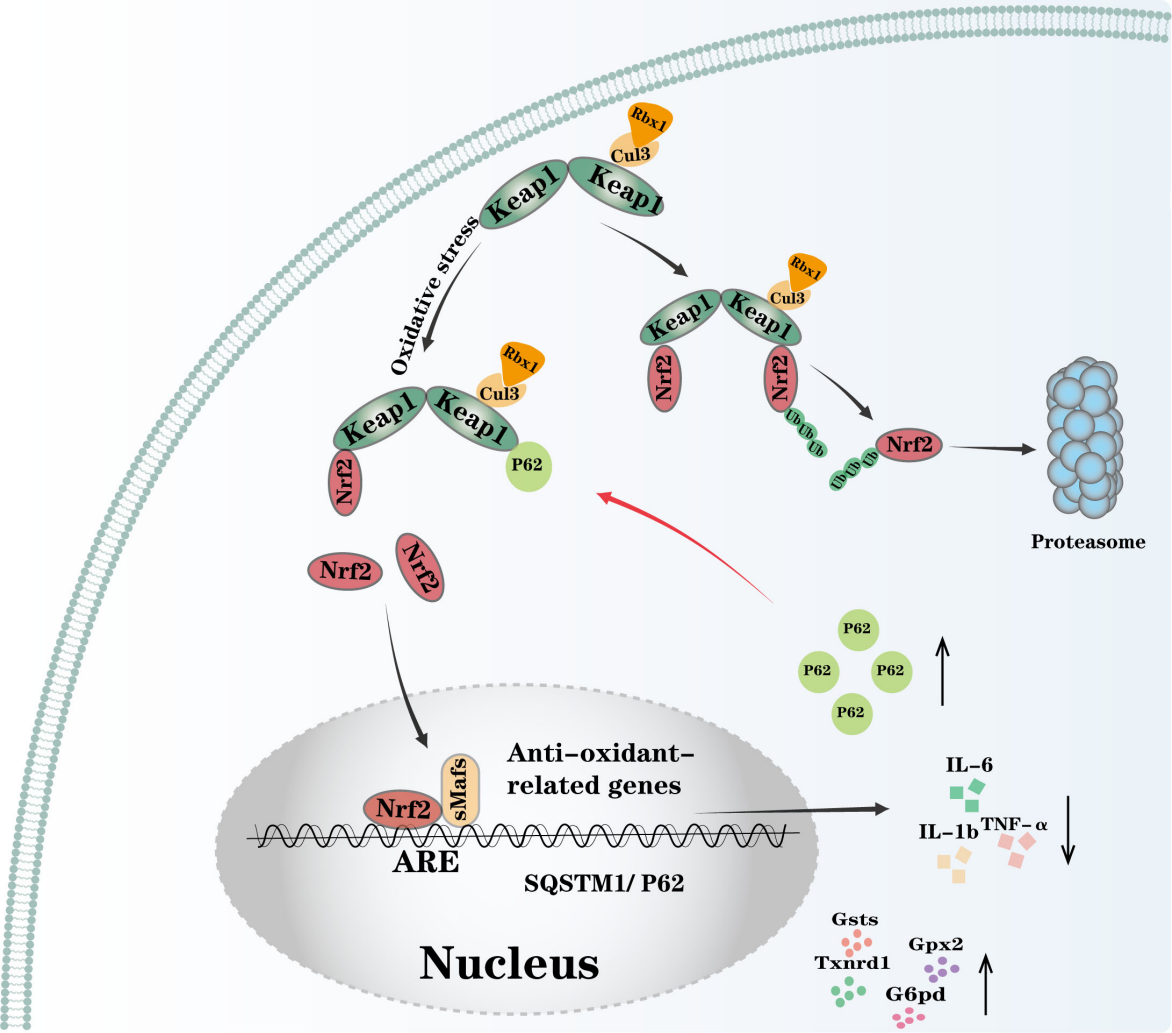
P62 activates Nrf2: Under basal conditions, two Keap1 molecules asymmetrically bind to cytoplasmic Nrf2, forming a complex with the E3 ubiquitin ligase Cul3/Rbx that promotes Nrf2 ubiquitination and proteasomal degradation, thereby maintaining low Nrf2 levels. Under oxidative stress, conformational changes in Keap1 inhibit Cul3/Rbx E3 ligase activity, allowing newly synthesized Nrf2 to escape Keap1 binding, translocate to the nucleus, induce antioxidant and anti-inflammatory gene expression, and suppress proinflammatory cytokines such as IL-1β, IL-6, and TNF-α. P62, a transcriptional target of Nrf2, is upregulated in this process. Elevated cytoplasmic P62 further binds Keap1, preventing Nrf2 degradation and amplifying Nrf2 activation through a positive feedback loop. Gsts, glutathione S-transferases; Gpx2, glutathione peroxidase 2; Txnrd1, thioredoxin reductase 1; G6pd, glucose-6-phosphate dehydrogenase; sMafs, small Maf proteins; ARE, antioxidant response element.

Nrf2 (nuclear factor erythroid 2-related factor 2) is a master regulator of cellular responses to environmental stress. As a central mediator of cytoprotective enzyme induction, Nrf2 drives the expression of a wide range of detoxifying and antioxidant enzymes, including glutathione S-transferases, glutathione peroxidase 2, thioredoxin reductase 1, and glucose-6-phosphate dehydrogenase. Simultaneously, Nrf2 suppresses the induction of pro-inflammatory cytokine genes; for example, it promotes the expression of heme oxygenase-1 (HO-1) to inhibit NF-κB activation induced by oxidative stress and directly antagonizes the induction of pro-inflammatory genes such as IL-6, IL-1β, and TNF-α. Through these mechanisms, Nrf2 orchestrates broad cytoprotective and anti-inflammatory responses (**Figure 4**) (80, 81). The intracellular abundance of Nrf2 is tightly regulated by KEAP1. As an adaptor protein for Cullin-3-based ubiquitin ligase complexes, Keap1 forms an asymmetric homodimer that binds to Nrf2 under basal, non-stress conditions. This interaction recruits the E3 ubiquitin ligase complex Cul3/Rbx1, which mediates Nrf2 ubiquitination and subsequent proteasomal degradation. This constitutive degradation process maintains low cellular levels and activity of Nrf2 under homeostatic conditions (82, 83). Nrf2 activation occurs through both canonical and non-canonical pathways. In the canonical pathway, several redox-sensitive cysteine residues on Keap1 respond to elevated ROS. Under oxidative stress, conformational changes in Keap1 impair the activity of its associated Cul3-based E3 ligase, preventing newly synthesized Nrf2 from binding Keap1. As a result, Nrf2 escapes ubiquitination, translocates into the nucleus, and activates the expression of target genes (83, 84). In the non-canonical pathway, p62 competes with Nrf2 for Keap1 binding via its KIR motif, leading to Keap1 ubiquitination and degradation (78, 85). This competitive interaction releases Nrf2 from Keap1, allowing Nrf2 to accumulate and translocate into the nucleus, where it dimerizes with small Maf proteins (sMafs). The Nrf2–sMaf heterodimer binds to antioxidant response elements (AREs) or electrophile response elements in the regulatory regions of cytoprotective genes, driving transcription and activating a broad spectrum of cellular defense processes (82, 86). Notably, p62 itself is also a transcriptional target of Nrf2, establishing a positive feedback loop in which nuclear Nrf2 accumulation enhances p62 expression, and p62, in turn, further stabilizes Nrf2 (**Figure 4**) (14, 15).

In the short term, this mechanism is beneficial for suppressing inflammation. However, as Nrf2 also plays a critical role in metabolic reprogramming, its sustained activation can divert glucose and glutamine into anabolic pathways, promote purine biosynthesis, and modulate the pentose phosphate pathway (PPP) to support cell proliferation (87). This metabolic rewiring offers a fundamental advantage for the growth and survival of cancer cells (88). Furthermore, Nrf2 directly regulates apoptosis. In mammalian cells, Nrf2 transcriptionally upregulates the anti-apoptotic proteins Bcl-2 and Bcl-xL, thereby promoting resistance to apoptosis and enhancing the tumorigenic capacity of cancer cells (89, 90).

### 3.4 P62 indirectly suppresses the production of IL-6

The cytokine IL-6, produced by immune cells, is both a key inflammatory mediator and a critical pro-tumorigenic factor. In CRC, the IL-6/STAT3 signaling pathway plays a pivotal role in inflammation-driven carcinogenesis. This pathway is aberrantly activated in CRC and contributes to nearly all stages of tumor progression, including cancer cell growth, proliferation, angiogenesis, invasion, and metastasis (52, 91). p62 indirectly regulates IL-6 synthesis via the mTOR pathway. Specifically, p62 interacts with Raptor and the E3 ubiquitin ligase TRAF6, which induces K63-linked ubiquitination of mTOR. This p62–TRAF6-dependent ubiquitination event activates mTOR, which, through the mTORC1/c-Myc axis, regulates stromal glucose and amino acid metabolism, modulating cellular redox balance and ROS production, thereby influencing IL-6 synthesis (20, 92). While this mechanism contributes to anti-inflammatory effects, its sustained activation may also provide survival and metabolic advantages to cancer cells, highlighting the dual role of p62 in CRC (92).

## 4 P62 activates cancer cell growth signaling and forms a positive feedback loop, leading to its further intracellular accumulation

### 4.1 Activation of NF-κB°

The NF-κB (nuclear factor κB) family of transcription factors consists of five distinct DNA-binding proteins: RelA, c-Rel, RelB, NF-κB1 (p50 and its precursor p105), and NF-κB2 (p52 and its precursor p100). These members typically exist in the cytoplasm as inactive hetero- or homodimers, associated with inhibitory proteins called IκBs, or, in the case of NF-κB1 and NF-κB2, in their unprocessed precursor forms. Upon stimulation by various signals, including those from antigen receptors, pattern-recognition receptors, and cytokine receptors such as TNF-α and IL-1, NF-κB dimers undergo differential activation, translocating to the nucleus where they bind DNA and regulate the transcription of a wide range of target genes (93, 94). NF-κB plays a central role in regulating inflammation and immune responses, with its activation reflecting the progression of inflammatory processes (95). Notably, NF-κB is involved in almost every stage of tumor development, establishing it as a critical molecular link between inflammation and cancer (96).

**Figure 5:**
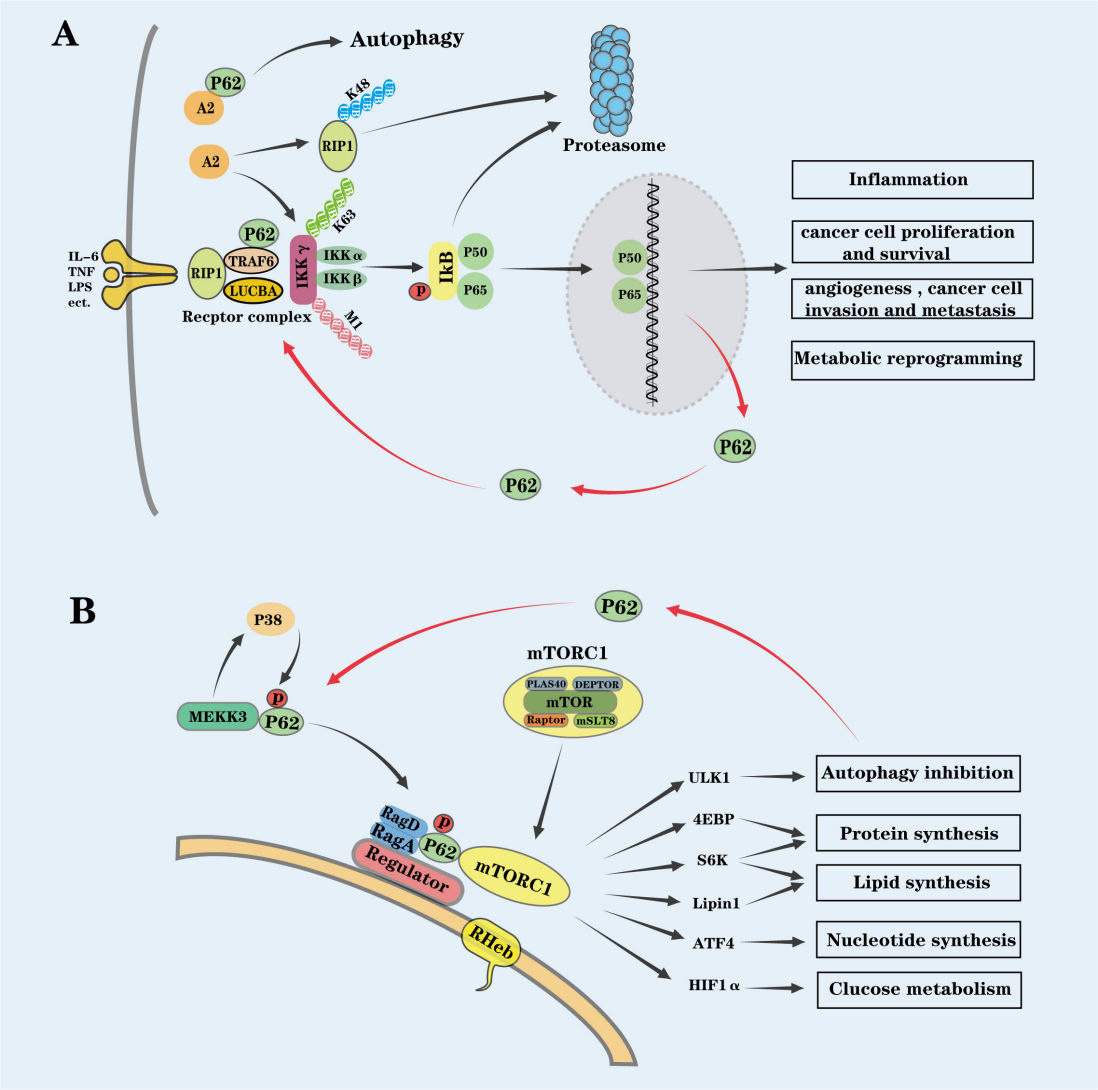
P62 activates oncogenic NF-κB and mTORC1 pathways. **(A)** NF-κB Activation: NF-κB dimers, bound to IκB inhibitors in the cytoplasm, are released upon IKK complex (IKKα, IKKβ, and IKKγ) activation by receptors like TNF-α and IL-1. Activated IKK phosphorylates IκB, leading to its degradation, and allows NF-κB dimers to translocate to the nucleus, promoting inflammation and tumorigenesis. Ubiquitin-editing enzyme A2 inhibits NF-κB activation by reversing K63-linked ubiquitination of IKKγ and promoting K48-linked ubiquitination and proteasomal degradation of the kinase RIP1. P62 also promotes the autophagic degradation of A2. As an NF-κB target gene, P62 forms a feedback loop to sustain its own accumulation and NF-κB activation. **(B)** mTORC1 Activation: mTORC1 comprises the core kinase mTOR, the substrate recruiter Raptor, the stabilizer mLST8, and two inhibitory subunits, PRAS40 and DEPTOR. Under high amino acid conditions, MEKK3 phosphorylates P62 by its downstream kinase p38δ. Phosphorylated P62 binds Raptor, the pentameric Regulator complex and Rag GTPases (heterodimers of RagA or RagB with RagC or RagD), promoting mTORC1 activation at the lysosomal membrane. Phosphorylated P62 also recruits TRAF6 to mTORC1, leading to K63-linked polyubiquitination and activation. mTORC1 inhibits ULK1, reducing autophagy and increasing P62 accumulation, which further stimulates mTORC1, establishing a positive feedback loop. Activated mTORC1 supports tumor growth by regulating biosynthesis pathways (lipids, proteins, nucleotides) and represses autophagy to prevent P62 degradation. Together, P62-driven activation of NF-κB and mTORC1 plays a key role in tumor progression by linking inflammation, metabolic reprogramming, and autophagy disruption.

The NF-κB signaling pathway is activated through two primary routes: the canonical pathway, which is rapidly induced by pro-inflammatory cytokines and antigens, and the non-canonical pathway, which requires *de novo* synthesis of NF-κB-inducing kinase (NIK). In both pathways, the IKK complex serves as the central activating factor. The IKK complex consists of three subunits: the catalytic subunits IKKα and IKKβ, and the regulatory subunit IKKγ. In the canonical pathway, receptor engagement by antigens, TNF-α, or IL-1 recruits a series of E3 ubiquitin ligases to form receptor complexes that activate the IKK complex. Activated IKK phosphorylates the inhibitor IκB, leading to its K48-linked polyubiquitination by E3 ubiquitin ligase complexes and subsequent proteasomal degradation. This degradation releases NF-κB heterodimers, allowing their translocation to the nucleus and the initiation of target gene transcription (93). Activation of the IKK complex requires trans-autophosphorylation between two adjacent catalytic subunits, a process mediated by the bridging of two IKK complexes through ubiquitin chains attached to the IKKγ subunit (96). This non-degradative ubiquitination of IKKγ involves K63- and M1-linked ubiquitin chains, catalyzed by E3 ligases TRAF6 and Linear Ubiquitin Chain Assembly Complex (LUBAC), and is facilitated by the interaction between p62 and TRAF6 (97, 98). The deubiquitinating enzyme A2 can counteract the K63-linked ubiquitination of IKKγ and promote K48-linked ubiquitination of Receptor-Interacting Protein Kinase 1 (RIP1), leading to its proteasomal degradation. These actions weaken the recruitment and activation of the IKK complex, thereby suppressing NF-κB activation (99). A2 is a direct target of p62, which promotes its autophagic degradation, thereby enhancing NF-κB activation (100). During chronic intestinal inflammation, immune cells infiltrating the gastrointestinal mucosa secrete pro-tumorigenic cytokines, including TNF-α, IL-1, and IL-17, as well as ROS, all of which further enhance NF-κB activity (**Figure 5A**) (93, 95).

The NF-κB signaling pathway is involved in nearly every aspect of CRC development (51). Initially, as part of the inflammatory cascade, NF-κB induces and maintains a chronic inflammatory microenvironment. It drives the polarization of tumor-associated macrophages (TAMs) from the M1 to the M2 phenotype, characterized by reduced tumoricidal activity, enhanced angiogenesis, and tissue remodeling, while simultaneously suppressing antitumor immune responses (96). Additionally, NF-κB promotes the expression of anti-apoptotic genes, such as the caspase-8 inhibitor FLIP, apoptosis inhibitors c-IAP1/2 and XIAP, and members of the Bcl-2 family, facilitating cell proliferation and inhibiting apoptosis. Furthermore, NF-κB stimulates tumor angiogenesis by regulating vascular endothelial growth factor (VEGF) and other angiogenic factors, including basic fibroblast growth factor (bFGF), IL-8, and matrix metalloproteinase-9 (MMP-9), all of which support cancer cell invasion and metastasis (101). Finally, NF-κB plays a role in metabolic reprogramming in tumor cells, for example, by upregulating PD-L1 and SCO2 (102, 103). Notably, p62 has been identified as a downstream target gene of NF-κB. NF-κB activation upregulates p62 expression, leading to its intracellular accumulation, which in turn further activates NF-κB, establishing a positive feedback loop that sustains p62 buildup (**Figure 5A**) (16).

### 4.2 Activation of mTOR

The mechanistic target of rapamycin (mTOR) is a 289 kDa serine/threonine kinase belonging to the phosphatidylinositol 3-kinase–related kinase (PIKK) family, acting as a central coordinator of extracellular signals and intracellular status. mTOR is the catalytic subunit of two distinct multiprotein complexes: mTOR complex 1 (mTORC1) and mTOR complex 2 (mTORC2). Among these, mTORC1 serves as a critical intracellular sensor for nutrients and energy, integrating signals from growth factors, cellular energy status, amino acids, and oxygen availability to regulate cell growth, proliferation, metabolism, and autophagy. The mTORC1 complex includes core components such as mTOR, the substrate-binding protein Raptor, the stabilizing protein mLST8, and two inhibitory subunits, PRAS40 and DEPTOR (104). Under normal conditions, mTORC1 promotes anabolic processes like protein, lipid, and nucleotide synthesis, while inhibiting catabolic processes like autophagy to drive cell growth. These metabolic pathways are activated through phosphorylation of downstream effectors. For instance, phosphorylation of p70 S6 kinase 1 (S6K1) and eukaryotic initiation factor 4E binding protein (4EBP) enhances mRNA translation and protein synthesis; phosphorylation of S6K1 and the cofactor Lipin 1 activates sterol regulatory element-binding proteins (SREBPs), stimulating lipid biosynthesis; induction of ATF4-dependent MTHFD2 expression promotes nucleotide synthesis; and increased translation of the transcription factor HIF-1α alters glucose metabolism. Conversely, phosphorylation of ULK1, a key autophagy-initiating kinase, by mTORC1 inhibits autophagy, reinforcing anabolic metabolism (**Figure 5B**) (23, 105).

Multiple signals, including growth factors, hormones, energy status, and amino acid availability, converge to regulate mTORC1 activity. In response to elevated amino acid levels, MEKK3 interacts with the PB1 domain of p62, enabling its downstream kinase p38δ to phosphorylate p62 at Thr269 and Ser272 (106). This phosphorylation is essential for amino acid–induced mTORC1 activation. Phosphorylated p62 directly associates with both Raptor, a core component of mTORC1, and Rag GTPases (heterodimers of RagA or RagB with RagC or RagD), which are anchored to the lysosomal membrane through the pentameric Ragulator complex (composed of MP1, p14, p18, HBXIP, and C7orf59). Upon amino acid stimulation, Rag GTPases undergo nucleotide exchange to adopt an active conformation, allowing their binding to Raptor and subsequent recruitment of mTORC1 to the lysosomal surface (107). At this site, mTORC1 encounters the small GTPase Rheb, which directly activates mTORC1 kinase activity (20). Additionally, phosphorylated p62 recruits the E3 ubiquitin ligase TRAF6 to the mTORC1 complex via its interaction with Raptor, leading to K63-linked polyubiquitination of mTOR, a modification essential for mTORC1 activation (38, 106). Thus, p62 plays a pivotal role in orchestrating mTORC1 activation. Additionally, mTORC1 suppresses autophagy by phosphorylating the autophagy-initiating kinase ULK1 (23, 105), reducing autophagic flux and causing p62 accumulation. Elevated p62 levels, in turn, further stimulate mTORC1 activity, establishing a positive feedback loop that sustains mTORC1 activation and inhibits autophagy (**Figure 5B**).

As a critical regulator of cell proliferation, metabolism, and growth, mTORC1 functions as a downstream effector of multiple signaling cascades and plays an essential role in various cancers, including CRC (108). In CRC, mTORC1 contributes to tumorigenesis through its involvement in the PI3K/Akt/mTOR and Ras/Raf/MEK/ERK (MAPK) pathways. Activation of Akt promotes glucose uptake and glycolysis, supporting proliferative processes while suppressing cell death (109, 110). Similarly, activation of the MAPK pathway drives continuous proliferative signaling. Dysregulation of either pathway leads to uncontrolled tumor cell growth and resistance to apoptosis (109). Furthermore, AKT signaling enhances tumor progression by upregulating VEGF expression, promoting angiogenesis within the TME (110).

## 5 P62 and DNA damage repair

### 5.1 DNA single-strand repair

**Figure 6:**
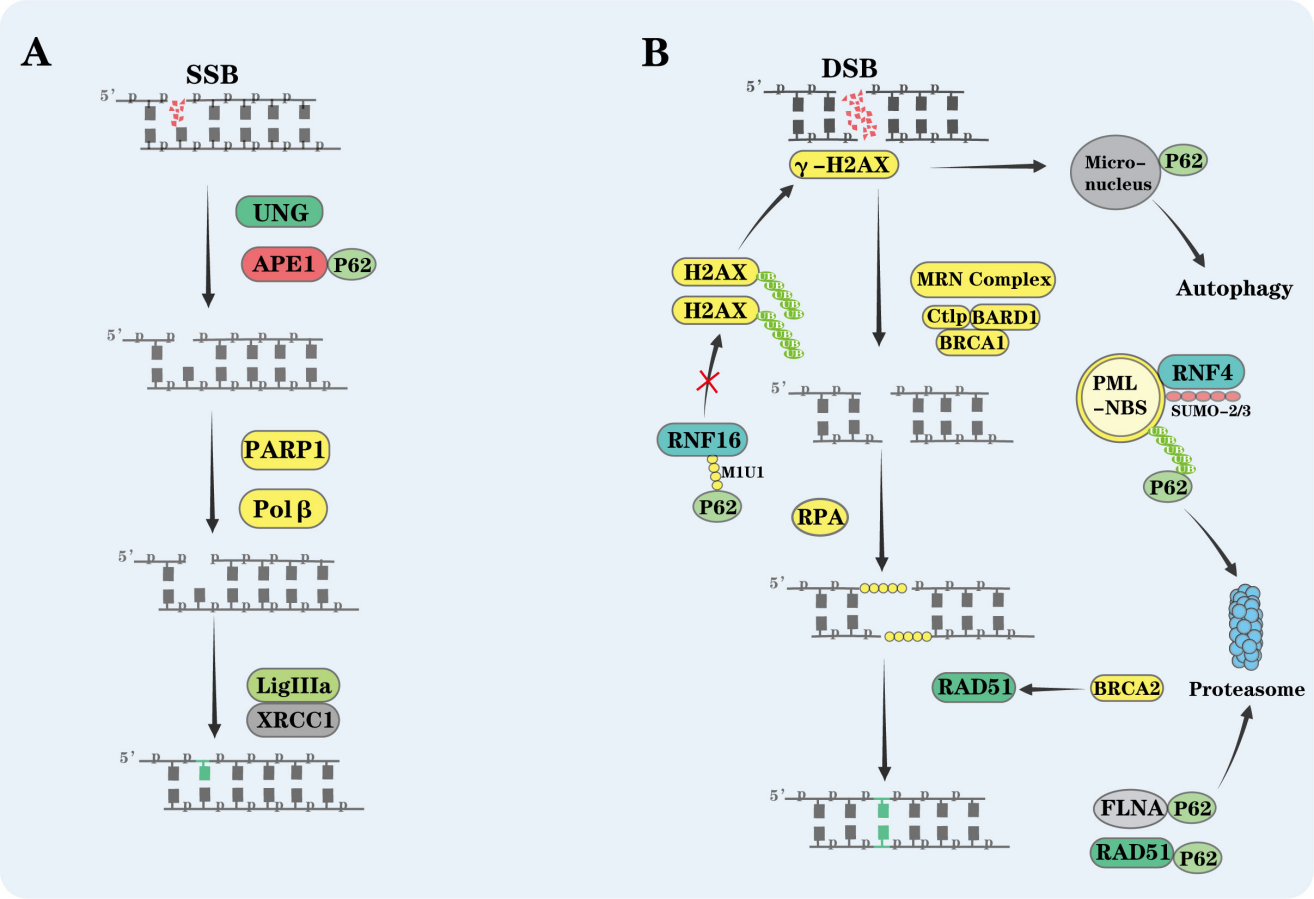
P62 inhibits DNA damage repair. **(A)** Impact on Base Excision Repair (BER): During base excision repair (BER), Uracil-DNA Glycosylase (UNG) recognizes and removes uracil from DNA, creating an AP site. The AP site is then cleaved by AP endonuclease 1 (APE1), and poly (ADP-ribose) polymerase 1 (PARP1) protects the resulting single-strand breaks (SSBs) and recruits repair factors. DNA polymerase β (Polβ) fills the resulting nucleotide gaps, while the XRCC1–Ligase IIIα (XRCC1–LigIIIα) complex seals the remaining breaks. **(B)** Inhibition of Homologous Recombination (HR): In homologous recombination (HR), Histone H2AX is phosphorylated at serine 139 (Ser139) at DNA double-strand break (DSB) sites, forming the DNA damage marker γ-H2AX, recruiting the MRE11-RAD50-NBS1 (MRN) complex and the BRCA1 DNA repair complex for DNA end processing. And the DNA repair protein BARD1 and the ubiquitin ligase–associated protein Ctlp assist in stabilizing this processing complex. Replication protein A (RPA) binds single-stra nded DNA (ssDNA) to prevent undesired interactions, The DNA repair protein BRCA2 directly binds the homologous recombination core protein RAD51, promoting the assembly of RAD51 nucleoprotein filaments on single-stranded DNA (ssDNA) to mediate the completion of DNA repair. P62 inhibits HR by targeting γ-H2AX-containing micronuclei for autophagic degradation. It also reduces HR repair by promoting proteasomal degradation of promyelocytic leukemia nuclear bodies (PML-NBs), which are enriched with HR proteins. Additionally, P62 suppresses HR by inhibiting RNF168 activity and promoting degradation of RAD51 and filamin A (FLNA), thereby reducing recruitment of HR factors.

DNA single-strand breaks (SSBs) are among the most common forms of DNA damage, typically caused by oxidative stress or abnormal enzymatic activity. Rapid repair of these breaks is essential to prevent their progression into more harmful DSBs. Base excision repair (BER) is the primary mechanism for addressing endogenous DNA damage, such as oxidation, alkylation, and deamination, as well as simple SSBs. BER occurs in three stages: damage recognition and base excision, incision at apurinic/apyrimidinic (AP) sites, and subsequent repair of the DNA (**Figure 6A**) (111, 112). The process is a coordinated, sequential series involving over 30 proteins, with SSBs forming intermediates during the repair process (113). p62 primarily regulates the second step—incision at AP sites.

Specifically, p62 undergoes acetylation at lysine 264 by the acetyltransferase hMOF, promoting its nuclear accumulation and enhancing its interaction with apurinic/apyrimidinic endonuclease 1 (APE1), the key enzyme in the BER pathway. APE1 cleaves the phosphodiester bond at AP sites, creating an SSB and enabling subsequent repair by DNA polymerases and ligases (114, 115). Nuclear-enriched p62 binds to the C-terminal region of APE1, facilitating its recruitment to damaged sites and thereby enhancing BER (**Figure 6A**) (116). Beyond BER, p62 also contributes to nucleotide excision repair (NER). As a core component of the NER machinery, transcription factor IIH (TFIIH) includes the helicase subunit XPD. Evidence suggests that p62 interacts with the structural subunit p44 of TFIIH, enhancing the DNA-binding affinity of XPD and its helicase activity. This interaction ultimately improves the efficiency of NER (117).

These findings highlight p62’s essential role in facilitating single-strand DNA repair through its contributions to both the BER and NER pathways.

### 5.2 DNA double-strand break repair

DSBs, which disrupt both strands of the DNA helix, are among the most severe forms of DNA damage. Unrepaired or misrepaired DSBs can lead to cell death or chromosomal rearrangements, such as deletions and translocations, thereby contributing to genomic instability and promoting carcinogenesis more readily than SSBs. In eukaryotic cells, DSBs are primarily repaired through two major pathways: homologous recombination (HR) and non-homologous end joining (NHEJ) (118).

In contrast to its supportive role in SSB repair, nuclear p62 negatively regulates DSB repair, primarily through autophagy-dependent mechanisms that target key proteins involved in HR. For example, p62 regulates the stability and proteasomal degradation of DNA repair factors, including the homologous recombination core protein RAD51, the E3 ubiquitin ligase RNF4, and the histone variant H2AX (**Figure 6B**). H2AX, a variant of histone H2A, plays a central role in the DNA damage response (119–122).

Upon the occurrence of DSBs, PI3K-like serine/threonine protein kinases (PIKKs), including ATM, ATR, and DNA-PK, rapidly phosphorylate histone H2AX at serine 139, thereby generating the DNA damage marker γ-H2AX. This phosphorylated form serves as a critical damage marker that recruits DNA repair proteins and facilitates the assembly of DNA damage foci (123). However, p62 selectively targets micronuclei containing phosphorylated γ-H2AX for autophagic degradation. While this activity contributes to genome stability by removing damaged nuclear material, it simultaneously suppresses the DNA repair process, particularly HR-mediated repair (**Figure 6B**). Thus, p62 functions as a negative regulator of DSB repair, highlighting its dual role in genome maintenance (119).

PML nuclear bodies (PML-NBs) are highly dynamic, SUMO-enriched, membraneless nuclear structures, composed of an outer scaffold formed by the core protein PML and inner components, including the DNA repair complex Mre11, the DNA helicase WRN, and the DNA helicase BLM. PML-NBs are involved in regulating the HR process of DNA repair (124). RNF4, an E3 ubiquitin ligase belonging to the SUMO-targeted ubiquitin ligase (STUbL) family, participates in the ubiquitination and subsequent proteasomal degradation of PML. Like ubiquitin, SUMO-2 and SUMO-3 can form polymeric SUMO chains through conserved lysine residues (e.g., Lys11). These SUMO-2/3 chains attached to PML act as recruitment signals for RNF4. With its four closely spaced SUMO-interacting motifs (SIMs), RNF4 recognizes SUMOylated PML and catalyzes the assembly of ubiquitin chains on PML. Following this modification, p62 mediates the proteasomal targeting and degradation of PML nuclear bodies (**Figure 6B**) (120, 125).

Ubiquitination plays a pivotal role in DNA damage repair. The ubiquitination cascade induced by DNA DSBs begins with RNF8-dependent conjugation of ubiquitin to histone H1. E3 ligase RNF8 then recruits another E3 ligase, RNF168, which catalyzes the formation of Ub-K63 chains on histones H2A and H2AX at lysines 13–15. This regulatory ubiquitination is essential for recruiting downstream mediators in the DSB response pathway, including the DNA damage response protein 53BP1 and the DNA repair complex BRCA1 (126, 127). The MIU1 motif of RNF168 is critical for its interaction with p62. By binding to this MIU1 motif, p62 may directly inhibit the E3 ligase activity of RNF168, reducing the recruitment of key HR repair factors such as RAD51, RAP80 and BRCA1, ultimately suppressing the HR repair process (**Figure 6B**). Additionally, this RNF168-dependent ubiquitination reaction is pivotal for the NHEJ pathway, suggesting that p62 also regulates this repair process (37, 121).

Filamin A (FLNA), an actin-binding protein, crosslinks actin filaments into dynamic three-dimensional structures and functions as a scaffold in various signaling networks. Studies show that FLNA interacts with BRCA1/2 and recruits the HR protein RAD51 to facilitate DNA damage foci formation. FLNA is also necessary for efficient interaction between HR-related proteins DNA-PKcs and Ku86 (128, 129). p62 promotes the proteasomal degradation of FLNA and RAD51, reducing nuclear RAD51 levels and delaying DNA repair (**Figure 6B**) (122).

While CRC is primarily driven by sporadic factors, approximately 10% of cases are linked to hereditary gene mutations. Mutations in tumor suppressor genes, oncogenes, DNA mismatch repair genes, alterations in DNA methylation, and dysregulated signaling pathways are major drivers of CRC progression (130). During the persistent development of CRC-associated inflammation, infiltrating inflammatory cells in intestinal tissue generate reactive oxygen and nitrogen species (ROS and RNS), which induce tissue and DNA damage (131). However, due to the inhibitory effect of p62 on DSB repair, the cell’s intrinsic DNA repair capacity is compromised, leading to the accumulation of damaged DNA, genomic instability, and subsequent gene mutations.

## 6 Conclusion

In conclusion, p62 plays a dual role in CRC development, particularly in colitis-associated CRC (CA-CRC). In the early stages of CRC-related inflammation, P62 acts as an autophagy adaptor to suppress tumorigenesis by promoting the degradation of damaged organelles, inflammasome components, and inflammatory mediators (12, 13). However, under prolonged inflammatory stimulation, positive feedback loops between p62 and signaling factors like Nrf2, mTOR, and NF-κB lead to its excessive intracellular accumulation, at which point its function is reversed (14, 16, 20, 78, 97, 98, 132). High intracellular levels of p62 interact with these signaling pathways to indirectly promote metabolic reprogramming, activate proliferative signals, enhance resistance to apoptosis, and stimulate angiogenesis. Additionally, nuclear aggregation of p62 directly impairs DNA damage repair, leading to the accumulation of DNA lesions (26). Collectively, these effects create a foundation for CRC initiation, transforming p62 from a tumor suppressor into a tumor promoter. Thus, eliminating the excessive accumulation of p62 during chronic inflammation is crucial for preventing CRC initiation and suppressing its progression.

It is well-established that p62 relies on its LIR and UBA domains to function as an autophagic cargo receptor, with autophagy being its primary mode of intracellular degradation. Autophagy inducers represent one of the potential strategies for modulating p62 protein levels and restoring autophagic flux in CRC, with promising preclinical therapeutic implications. Accumulating evidence from CRC cell lines and xenograft models demonstrates that pharmacological activation of autophagy promotes autophagic flux and facilitates p62 degradation through distinct mechanisms. For instance, rapamycin and its analogs, as mTORC1 inhibitors, relieve suppression of the ULK1 initiation complex, thereby increasing LC3-II levels, reducing p62 accumulation, and inhibiting tumor cell proliferation (133). Metformin, through AMPK activation and subsequent inhibition of mTOR signaling, has similarly been shown to enhance autophagy and suppress tumor growth in CRC models (134). Natural autophagy inducers, including triptolide and resveratrol, have also been reported to upregulate the Beclin-1/LC3 axis, decrease p62 expression, and potentiate the cytotoxic effects of chemotherapeutic agents *in vitro* (135, 136). Nevertheless, autophagy induction carries inherent risks and limitations. While autophagy generally exerts tumor-suppressive effects during early tumorigenesis, excessive or sustained autophagy in established tumors may instead promote cancer cell survival, therapeutic resistance, and even metastatic progression (137). Furthermore, interpatient heterogeneity in genetic background and tumor microenvironment may substantially influence responsiveness to autophagy inducers, potentially leading to systemic adverse effects such as immunosuppression or metabolic disturbances. Therefore, future clinical translation will require precise and context-dependent modulation of autophagic activity to balance therapeutic efficacy with safety and to optimize p62-targeted strategies in CRC.
